# Gene Expression of Adhesion Molecules in Endothelial Cells from Patients with Peripheral Arterial Disease Is Reduced after Surgical Revascularization and Pharmacological Treatment

**DOI:** 10.1155/2013/412761

**Published:** 2013-02-27

**Authors:** Franca Marino, Luigina Guasti, Matteo Tozzi, Laura Schembri, Luana Castiglioni, Elisabetta Molteni, Gabriele Piffaretti, Patrizio Castelli, Marco Cosentino

**Affiliations:** ^1^Department of Clinical and Experimental Medicine, University of Insubria, Via Ottorino Rossi No. 9, 21100 Varese, Italy; ^2^Center of Research in Medical Pharmacology, University of Insubria, Varese, Italy

## Abstract

Atherosclerosis is an inflammatory disease characterized by immunological activity, in which endothelial dysfunction represents an early event leading to subsequent inflammatory vascular damage. We investigated gene expression of the adhesion molecules (AMs) ICAM-1, VCAM-1, and *β*1-integrin in endothelial cells (ECs) isolated from venous blood (circulating EC, cEC) and purified from femoral plaques (pEC) obtained from 9 patients with peripheral artery disease (PAD) submitted to femoral artery thrombendarterectomy (FEA). In addition, in peripheral blood mononuclear cells (PBMCs) of the same subjects, we investigated gene expression of IFN-*γ*, IL-4, TGF-*β*, and IL-10. Patients were longitudinally evaluated 1 month before surgery, when statin treatment was established, at the time of surgery, and after 2 and 5 months. All AM mRNA levels, measured by means of real-time PCR, in cEC diminished during the study, up to 41–50% of initial levels at followup. AM mRNA expression was significantly higher in pEC than in cEC. During the study, in PBMCs, TGF-*β* and IL-10 mRNA levels remained unchanged while IFN-*γ* and IL-4 levels increased; however, the ratio IFN-*γ*/IL-4 showed no significant modification. In PAD patients, FEA and statin treatment induce a profound reduction of AM expression in cEC and affect cytokine mRNA expression in PBMCs.

## 1. Introduction

Atherosclerosis (ATH) is an inflammatory disease characterized by intense immunological activity [1] that involves the formation of lesions in the arteries and is characterized by lipid accumulation, inflammation, cell death, and fibrosis. Recruitment of monocytes and lymphocytes from the peripheral blood to the intima of the vessel wall represents the primary event in atherogenesis. Among the factors involved in ATH, the local presence of high amounts of low-density lipoproteins (LDL) has a key role [2]. Healthy endothelium is an important barrier to the free passage of LDL and immune cells to the underlying interstitium, and dysfunction of endothelial cells (EC) is considered an early step in atherogenesis. EC dysfunction results in increased activation of these cells [3], sustained by upregulation of adhesion molecules (AMs) such as vascular cell adhesion molecule-1 (VCAM-1) and intercellular adhesion molecule-1 (ICAM-1). VCAM-1 expression precedes lesion formation and correlates positively with the extent of exposure to plasma cholesterol [4]. Cells can then change their shape and migrate at the surface of the endothelium to reach the junctions. Recently, we have confirmed the occurrence of extensive alteration of endothelial layers in surgical specimens of patients submitted to carotid endarterectomy [5, 6]. In view of the role played by EC in the development, progression, and clinical complications of ATH, reduction of their activation represents a key point in the prevention of atherosclerotic plaque progression [7].

Lower-extremity peripheral artery disease (PAD) is a chronic arterial disease of atherosclerotic origin characterized by the occurrence of vascular obstruction in various districts. Although public awareness of PAD is extremely low [8], this condition is related to a very high short-term incidence of myocardial ischemia, stroke, and death [9]. Besides an aggressive treatment of cardiovascular risk factors, common femoral artery thrombendarterectomy (FEA) is among the treatments for PAD and represents a safe procedure in PAD with acceptable perioperative morbidity [10], as plaque debulking is considered safer than percutaneous transluminal angioplasty [11, 12]. Moreover, surgery treatment with a statin, aimed at reaching low LDL-cholesterol levels, is highly recommended [10].

No information exists so far about the modification of inflammatory parameters in PAD patients after surgery and/or pharmacological treatment. The first aim of the present study was therefore to investigate adhesion molecules expression in endothelial cells (ECs) of patients with PAD submitted to peripheral artery revascularization associated with pharmacological treatment. To this end, we longitudinally measured ICAM-1, VCAM-1, and *β*1-integrin mRNA expression in EC isolated from venous blood (circulating EC, cEC) and purified from femoral plaques (pEC). In addition, as a secondary aim, we investigated in the same patients the expression of pro- and anti-inflammatory cytokines in peripheral blood mononuclear cells (PBMCs), in view of the pivotal role played by immune cell-derived cytokines in endothelial activation [13].

## 2. Materials and Methods

### 2.1. Subjects

We enrolled 9 patients (2 F, 7 M) with severe PAD scheduled for FEA and sent to the Lipid Clinic (Research Center on Dyslipidemia, University of Insubria, Varese, Italy) by the Department of Surgical and Morphological Sciences of our University Hospital. Only patients, who did not receive statins or steroids and had no ongoing clinical infection and/or the presence of infections in the previous three months, were enrolled. No patient had clinical contraindication for statin treatment. Clinical characteristics of the patients at enrollment are shown in Table 1.

After inclusion in the study, atorvastatin was prescribed to patients, according to established guidelines to reach recommended LDL-cholesterol levels [10] (10 mg/day to 4 patients with LDL <130 mg/dL, and 80 mg/day to 5 patients with LDL >130 mg/dL).

Patients were longitudinally studied at the following:

- the time of the decision for surgery before the institution of statin therapy (visit 1);
- the day of surgery (visit 2) after at least 1 month of statin therapy, before pharmacological preparation for the surgical procedure;
- 2 months (visit 3)
- 5 months (visit 4) after surgery.

At each visit, a sample of peripheral venous blood was obtained for clinical evaluation and for PBMC and cEC isolation. AM mRNA expression on cEC was evaluated at each visit, whereas cytokine mRNA expression in PBMC was analyzed at visits 1 and 4. In addition, at visit 2, after surgery the femoral plaque specimens were collected and subsequently processed to isolate pEC (see below).

The study conforms to the principles outlined in the Declaration of Helsinki for use of human tissues or subjects and the study protocol was approved by the local Ethics Committee. All patients gave a written informed consent.

### 2.2. Endothelial Cells (ECs) Purification

Venous blood was used to isolate cEC by means of immunomagnetic cell sorting. To this end, venous blood samples were divided in aliquots of 5 mL and each aliquot was washed with 5 mL of PBS/BSA 0.1%. Antibodies targeted to CD146 (Dynal A.S., Oslo, Norway) were added to beads using a target-to-bead ratio 1 : 4. After 30 min incubation, beads were washed and incubated for 20 min with 5 mL of blood aliquots. Cells were isolated from the mixture by placing the test tube in a magnetic particle concentrator. Medium was removed and cells were washed three times.

For the isolation of ECs from plaque (pEC), surgical specimens were placed in a solution of EDTA 0.05 M for 5 min and then washed in PBS solution for 5 min. Subsequently, tissue was placed in HEPES containing collagenase type II 0.2%, elastase 0.01%, and trypsinase inhibitor 0.1% and incubated under gentle stirring at 37°C for 90 min. Digested tissue was washed twice with PBS, filtered through a 70-micron cell strainer, and finally pECs were removed from the tissue mixture by immunomagnetic sorting (as above described for cEC).

ECs isolated from venous blood or from tissue were stained with Ab anti-CD45 and Ab anti-CD31 (specific marker for PECAM-1, which is highly expressed on EC) and visualized by fluorescence microscopy in order to test the absence of CD45+ positive cells. Typical preparations of CD146-sorted cells were negative for CD45 labelling and positive for CD31 labelling, confirming that immune cells were not present in our sample after cell sorting. A counting chamber was used to enumerate cells, which were finally frozen at −80°C until analysis.

### 2.3. Isolation of Peripheral Blood Mononuclear Cells (PBMCs)

PBMCs were separated by Ficoll-Paque Plus density gradient centrifugation from whole blood as previously described [14]. Cells were washed two times in NaCl 0.15 M and finally resuspended in appropriate medium for subsequent experiments. Typical PBMC preparations contained about 80% lymphocytes and 16% monocytes, as assessed by flow cytometry. Cell viability, assessed by the trypan blue exclusion test, was always >99%. Isolated cells were finally stored at −80°C until real-time PCR assays.

### 2.4. RNA Isolation and Real-Time Polymerase Chain Reaction (PCR) Analysis

Total mRNA was extracted from 1 × 10^6^ cells by Perfect RNA Eukaryotic Mini kit (Eppendorf, Hamburg, Germany) and the amount of RNA extracted was estimated by spectrophotometry at 260 nm. Total RNA was reverse transcribed using the high-capacity cDNA Archive Kit (Applied Biosystems, Foster City, CA, USA) according to the manufacturer's instructions.

Real-time PCR was performed by means of an ABI prism 7000 apparatus (Applied Biosystems, Foster City, CA, USA) using the assay-on-demand kits. Cycles included one 2 min hold (50°C), one 10 min (95°C), and 40 cycles according to the following steps: 15 s at 95°C (denaturation) and 1 min at 60°C (annealing and extension). Threshold cycle values (Ct1) for the genes of interest were calculated, normalized to 18S RNA (Ct2) (housekeeping) content, and finally expressed as 2^−ΔCt^, where ΔCt = Ct2 − Ct1. Primers were obtained from Applied Biosystems (Foster City, CA, USA), and are shown in Table 2.

### 2.5. Statistical Analysis

Data are presented as means ± standard error of the mean (SEM). Comparisons between independent measures were performed using the Mann-Whitney *U* test and comparisons between dependent measures were performed using the Wilcoxon test. Assessment of the statistical significance of correlations was performed by linear regression analysis. A commercial software was used for calculations (GraphPad Prism version 5.00 for Windows, GraphPad Software, San Diego, CA, USA, http://www.graphpad.com/).

## 3. Results

### 3.1. Clinical Evaluation

As described in Table 1, in the group of patients enrolled, 5 were smokers, 8 had hypertension, 7 had hypercholesterolemia, and 2 were subject with alcohol *habitus*. No change occurred during the study in all the clinical parameters evaluated (e.g., blood pressure, BMI, and routine exams) or in *habitus* that could influence parameters measured (smoking, diet, etc.).

All the patients had clinical symptoms referred to PAD and had monolateral (5 patients) or bilateral (4 patients) claudication and showed severe stenosis at angiographic evaluation. Severity of symptoms was evaluated according to Rutherford classification system as detailed in Table 1. Pharmacological treatments (Table 1) remained unchanged during the study with the only exception of the institution of atorvastatin therapy at the time of enrollment.

As expected, atorvastatin treatment and FEA significantly affected the lipid profile and hs-CRP of all the patients (Table 3) and no patient, either on 10 or 80 mg/die statin underwent any adverse event during the follow-up period.

### 3.2. Adhesion Molecules mRNA Levels in EC

**Figure 1:**
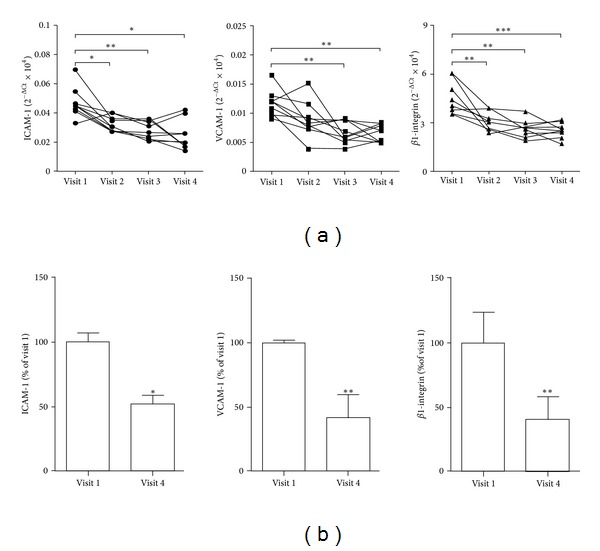
(a) ICAM-1 (circle), VCAM-1 (square), and *β*1-integrin (triangle) mRNA levels in cEC obtained from venous blood of PAD patients at the different visit times. (b) % of reduction of mRNA levels of ICAM-1, VCAM-1, and *β*1-integrin in cEC at visit 4 with respect to visit 1. *P < 0.05, **P < 0.01, and ***P < 0.001 versus visit 1.

As shown in Figure 1(a), mRNA levels of AM in cEC progressively decreased from visit 1 to visit 4. In particular, both ICAM-1 and *β*1-integrin were significantly reduced after 1 month, while reduction in expression for VCAM-1 was significant from visit 3. The % reduction for all molecule tested at visit 4 with respect to visit 1 varied from 41% to 50% (Figure 1(b)). No difference was observed in AM mRNA levels in EC between patients on atorvastatin 10 or 80 mg/day and no relationship was found between AM mRNA expression and lipid profile or hs-CRP (data not shown).

AM mRNA levels in pEC at visit 2 (day of surgery) were always detectable and were significantly higher in comparison to those found in cEC isolated at the same visit (Table 4); however, no direct correlation between cEC and pEC mRNA levels was found for VCAM-1 (*r* ^2^ = 0.44; *P* = 0.051), ICAM-1 (*r* ^2^ = 0.005; *P* = 0.564), or *β*1-integrin (*r* ^2^ = 0.08; *P* = 0.830).

No difference was observed in mRNA levels of AM in EC between patients on atorvastatin 10 or 80 mg/day (data not shown).

### 3.3. Cytokine mRNA Levels in PBMC

**Figure 2:**
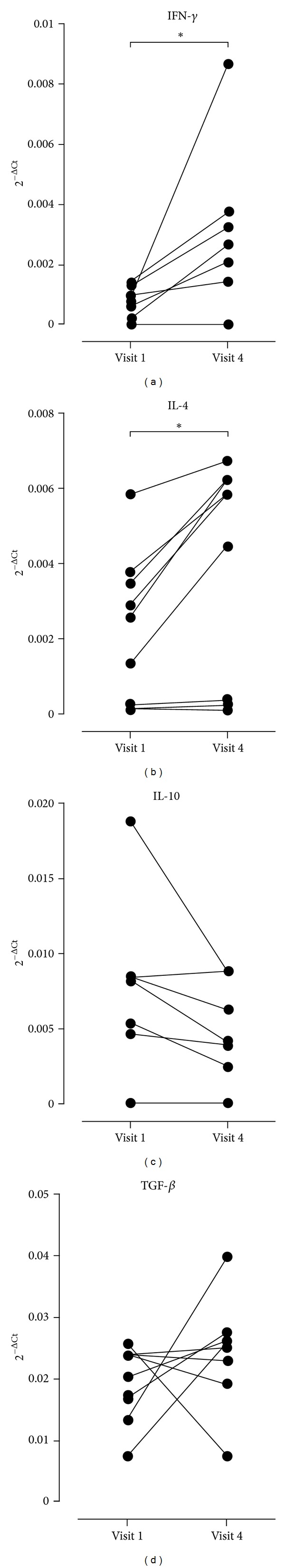
IFN-*γ*, IL-4, IL-10, and TGF-*β* mRNA levels in PBMCs obtained from venous blood of PAD patients at visit 1 and visit 4. * = *P* < 0.01 versus visit 1.

As shown in Figure 2, cytokine mRNA expression in isolated PBMC was significantly affected by FEA and statin treatment. In particular, a significant increase in mRNA expression from visit 1 to visit 4 was observed for IFN-*γ* and IL-4 while mRNA expression of IL-10 and TGF-*β* remained unchanged. No difference was, however, observed in the ratio IFN-*γ*/IL-4 (0,229 ± 0,083 at visit 1 versus 0,420 ± 0,151 at visit 4, *P* = 0,237).

No significant relationship was found between cytokine mRNA levels in PBMC and AM mRNA levels in either cEC or pEC as well as between the mRNA levels of the different cytokines in PBMCs and all clinical parameters measured through the study (data not shown).

## 4. Discussion

The main finding of this study is the observation that in PAD patients, surgery and pharmacological treatment induce in cEC a profound reduction of the mRNA levels of ICAM-1, VCAM-1, and *β*1-integrin. In addition, in circulating PBMCs of the same patients mRNA levels for IFN-*γ* and IL-4 increase and mRNA levels for TGF-*β* and IL-10 remain unchanged.

In patients with PAD, increased levels of soluble AM are associated with the presence, the severity, and the extent of ATH in the arteries of the lower limbs and are suggestive of a worse outcome [15]. Previous studies documented that PAD is associated with elevated levels of most inflammatory markers, such as CRP, CD40 ligand, fibrinogen, and others. Despite the potential benefits associated to the lowering of these molecule by therapeutic approaches, this is the first study showing that AM expression in cEC may be modified even in an advanced atherosclerotic stage. Our patients showed high levels of hs-CRP that were reduced 6 months after the beginning of the study. High hs-CRP level is commonly related to increased expression or circulating levels of different adhesion molecules [16] that can be considered as indicators of severity of peripheral ATH. Indeed, elevated levels of ICAM-1 in EC were found to be predictive of future development of PAD [17].

It has been shown that among drugs commonly used for managing cardiovascular disease, statins significantly improve endothelial function [18]. In particular a 4-week treatment with simvastatin was able to restore the vasodilator ability of endothelium, as assessed by increased vasodilatatory response to ACh, or contrasted L-NMMA-induced vasoconstriction, indicating that both stimulated and basal vasodilator function of the endothelium were enhanced [19]. The benefit of statin treatment in patients undergoing vascular surgery was documented by the reduced incidence of adverse cardiac outcome during the perioperative period and reduced adverse cardiac events after surgical procedures [20]. In our patients, statin treatment and surgical revascularization induced in cEC a decrease of mRNA expression of ICAM-1, VCAM-1, and *β*1-integrins (evident after 1 month of therapy and much more marked 5 months after surgery). We failed to observe any correlation between AM mRNA levels in cEC and pEC (with the marginal exception of a weakly significant positive correlation for ICAM-1). Possible explanations may include less sensitivity of pEC to the effects of pharmacological treatment and need much more time to observe an effect (as indirectly suggested by the additional reduction of AM mRNA levels in cEC after 2 and 5 months). Moreover, we cannot exclude that the small number of patients could have masked any possible correlation.

Statins may affect cellular function in advanced atherosclerotic stage, such as in patients scheduled for femoral artery revascularization and therefore affected by marked atherosclerotic lesions. For example, recently we reported that FEA associated with statin treatment in PAD patients was able to reduce in circulating polymorphonuclear leukocytes Angiotensin II (Ang II) type 1 receptor expression (a receptor pathway extensively involved in the proinflammatory and proatherogenic effects of Ang II) [21].

It is well recognized that inflammation as well as its related immunological events positively correlate with the severity of the PAD disease [22] and regulate all the stages of the inflammatory cascade, from endothelial activation to immune cell adhesion and subsequent remodeling of the vascular environment [23]. Cytokines are key glycoproteins which modulate all the aspects of vascular inflammation; moreover, cytokine balance is involved in plaque morphology and stabilization and in determining the structure of the fibrous cap. In cardiovascular diseases, cytokines are usually classified into two categories: proatherogenic and protective, but recently it has been shown that this classification is not exhaustive, because of the complexity and overlapping of the biological roles. For example IL-4, a cytokine classically produced by Th2 cells, has been proposed as protective in ATH, but IL-4 also increases the expression of P-selectin [24], VCAM-1, and matrix metalloprotease 1 and 12, which are implicated in aortic aneurysm formation and in acute coronary syndrome [25]. IFN-*γ*, a typical Th1 cytokine, is directly related to ATH by contributing to all the events that induce disease progression. IFN-*γ* is associated with increased levels of other proinflammatory cytokines, but it is also able to inhibit smooth muscle cells proliferation and collagen synthesis and to induce foam cells apoptosis within then lipid core, which could contribute to plaque stabilization resulting in beneficial effects in advanced stage of the disease [26]. In our patients' population, we observed that mRNA level of IL-4 and IFN-*γ* increased throughout the study. Since plaque destabilization has been related to reduced IL-4 values and increased level of IFN-*γ*, the increase of both IFN-*γ* and IL-4 might represent a protective effect [27]. No changes were observed in IL-10 and TGF-*β* mRNA expression. We cannot exclude that the absence of effects on IL-10 and TGF-*β* may be interpreted as the results of polytherapy differentially affecting specific lymphocyte subsets. In addition, it must considered that these two cytokines are preferentially produced by a specific T-lymphocyte subset (the regulatory subset that represents a low percentage of PBMCs) while in this study we have investigated mononuclear cells as a whole; another possible explanation of lack of effect on these cytokines is that possibly 6 months are not enough to observe any effect on these parameters.

We acknowledge that a critical point of this study was the low number of patients and the absence of untreated patients; this latter point in particular is, however, difficult to address, as it is clearly unethical to withdraw drug treatments or delaying therapy in such a kind of patients at extremely high risk for vascular acute events, even for limited periods. As for the limited number of subjects, it was underlined that the strict selection criteria (absence of statin treatment before the study) used for the study did not allow the inclusion of a greater number of patients. In our opinion, this study should be considered a pilot study that aimed to point the attention to cellular aspects in this particular group of patients in a late stage of the atherosclerotic process. Indeed, very few studies so far addressed the possibility to modulate EC and/or immune cell function in the later phases of plaque progression.

In conclusion, this study supports the hypothesis that also in patients with an advanced ATH status, reduction of endothelial dysfunction and modulation of the immune response may be achieved by a combination of surgical procedures and appropriate pharmacological therapy. Future studies should address the possible relationship of such effects with the long-term clinical outcome of patients.

## Figures and Tables

**Table 1:** Clinical characteristics of PAD patients at enrollment. Values are means ± SD.

PAD patients: 9	
Sex: 2 females (22,3%); 7 males (77,7%)	
Age, years: 68,0 ± 8,5	

Rutherford classification	
Stage 0–2: 0	
Stage 3: 3 (33,3%)	
Stage 4: 4 (44,4%)	
Stage 5: 2 (22,2%)	
Stage 6: 0	

Pharmacological treatment	
Antiplatelet drugs: 8 (88,8%)	
ACE inhibitors: 5 (55,5%)	
Ca^++^-channel blockers: 7 (77,7%)	
Hypoglycaemic drugs: 1 (11,1%)	
Other drugs: 7 (77,7%)	

Clinical notes	
Smokers: 5 (55,5%)	
Alcohol users: 2 (22,2%)	
Diabetes: 2 (22,2%)	
Hypertension*: 8 (88,8%)	
Hypercholesterolemia*: 7 (77,7%)	
Atrial fibrillation: 1 (11,1%)	
Ictus: 1 (11,1%)	

*Hypertension and hypercholesterolemia were defined as indicated by National Committee on Prevention, Detection, Evaluation, and Treatment of High Blood Pressure, National High Blood Pressure Education Program Coordinating Committee [8].

**Table 2:** Real-time PCR primers.

Gene symbol	UniGene ID	Interrogated sequence *RefSeq/GenBank mRNA *	Translate protein *RefSeq *	Exon boundary *RefSeq/GenBank mRNA *	Assay location *RefSeq/GenBank mRNA *	IMAGE clone ID *GenBank mRNA *	Amplicon length
ICAM-1	Hs.00277001_m1	NM_00201.2/BC015969.2	NP_000192.2	5-6/5-6	1502/1252	3506766	99
VCAM-1	Hs.00365486_m1	NM_001078.2/BC068490.1 o AK223266.1	NP_001069.1	7-8/7-8	1913/1012 o 1913	30339170	84
ITGB1	Hs.00559595_m1	NM_00222211.3/BC020057.1	NP_002202.2	11-12/11-12	1688/1532	3924411	102
IL-4	Hs.00174122_m1	NM_000589.2/BC066277.1 o BC067514.1	NP_000580.1	3-4/3-4	733/427 o 428	6971779 o 6971780	70
INFG	Hs.00174143_m1	NM_000619.2/BC070256.1	NP_000610.2	1-2/1-2	244/246	304146644	79
IL-10	Hs.00174086_m1	NM_000572/BC04252.1	NP_000563.1	3-4/3-4	440/437	40035920	119
TGFB1	Hs.99999918_m1	NM_004612.2/L11695.1	NP_004603.1	2-3/2-3	412/412	—	92

**Table 3:** Lipid, lipoprotein, and hs-CRP profile of patients during the study. Values are expressed as mean ± standard error (SEM). *P< 0,05 and **P< 0,01 versus visit 1.

	TC (mg/dL)	TG (mg/dL)	HDL-c (mg/dL)	LDL-c (mg/dL)	ApoA (mg/dL)	ApoB (mg/dL)	hs-CRP (mg/L)
Visit 1	243.6 ± 12.50	118.3 ± 15.28	57.00 ± 6.09	150.80 ± 877	185.10 ± 22.75	118.20 ± 6.20	4.24 ± 0.82
Visit 2	191.6 ± 15.44**	110.00 ± 14.52	52.2 ± 4.86	144.00 ± 9.91	179.30 ± 16.10	110.80 ± 10.12	—
Visit 3	170.08 ± 9.26**	115.10 ± 11.69	53.44 ± 4.36	86.80 ± 6.15*	165.80 ± 12.66	86.78 ± 5.26*	—
Visit 4	146.30 ± 10.35**	113.10 ± 10.94	52.33 ± 5.07	79.47 ± 5.07*	169.10 ± 15.92	78.44 ± 2.91**	2.12 ± 0.83*

TC: total cholesterol; TG: triglycerides; HDL-c: high-density lipoprotein cholesterol; LDL-c: low-density lipoprotein cholesterol; ApoA: apolipoprotein A; ApoB: apolipoprotein B; hs-CRP: high-sensitive C-reactive protein.

**Table 4:** Comparison between adhesion molecules mRNA levels in pEC and cEC at visit 2. Values are expressed as mean ± standard error (SEM).

Adhesion molecule	mRNA levels		*P *
	(2^−ΔCt^ × 10^4^)		
	pEC	cEC
ICAM-1	0.054 ± 0.013	0.032 ± 0.005	0.001
VCAM-1	0.013 ± 0.003	0.009 ± 0.003	0.026
*β*1-integrin	5.320 ± 1.160	3.100 ± 0.620	0.006
